# Ovariole number does not predict reproductive output or trade-off with immunity in *Drosophila melanogaster*

**DOI:** 10.1371/journal.pone.0333046

**Published:** 2025-10-14

**Authors:** Kiran Adhikari, Fajr Ali, Marco A. Malo Jr, Brian P. Lazzaro

**Affiliations:** Department of Entomology, Cornell University, Ithaca, New York, United States of America; Dartmouth Hitchcock Medical Center, UNITED STATES OF AMERICA

## Abstract

Reproduction and immunity are two energetically demanding traits that frequently trade-off with each other. Prior studies have suggested that the cost of reproduction limits the ability of *Drosophila melanogaster* females to fight bacterial infections, including the direct cost of developing and provisioning eggs. Ovariole number is a genetically variable trait in *Drosophila*, and is frequently assumed to be a good indicator of reproductive capacity. This implies ovariole number might also predict the quality of defense against bacterial infection. Here, we used 13 isogenic lines from the *Drosophila* Genomic Reference Panel to test whether reproductive investment trades off with immune response. These lines vary genetically for ovariole number, but we found ovariole number is not a predictor of reproductive output in infected females nor of resistance to bacterial infection. Furthermore, we saw no strong genetic tradeoff between fecundity and defense against infection in our experimental framework. Our results suggest that the evolutionary trade-off between reproduction and immunity could be weak, at least when measured in the laboratory, even when the physiological trade-off between these two traits is strong.

## Introduction

Organisms constantly need to balance their energy allocations across different physiological processes in order to sustain homeostasis, health, and evolutionary fitness. Immune defense and reproductive investments are two energetically demanding processes that often trade off with each other, such that higher investment in either leads to decline in quality of the other. Individuals in natural populations may be genetically variable for either or both traits, and trade-offs can be empirically observed as negative genetic correlations between them [1,2]. Even though reproduction-immunity trade-offs exist in multiple systems [2–4], the magnitude of these trade-offs varies across experiments and approaches to measurement. For example, studies within *Drosophila melanogaster* have shown that there can either be strong costs of mating on immunity [5], relatively very little direct trade-offs between reproduction and immunity [6], or intermediate and genetically variable relationships between the traits [7]. The variability across studies may stem from genetic differences between the populations being examined, difference in experimental conditions across laboratories, different choices of traits to be measured, or other reasons. In the present study, we leveraged natural genetic variability in ovary structure and egg production to test the hypothesis that reproductive investment is a direct cost that limits resistance to infection in *D. melanogaster*.

Reproductive and immune systems can be tightly interrelated in insects and the severity of infection can be influenced by the degree of reproductive activity [4]. For example, while mating is usually immunosuppressive in *D. melanogaster* [7–9], mated females lacking a germline resist bacterial infection at levels comparable to unmated females with intact germline [10–13]. Similarly, mutant *D. melanogaster* that arrest investment in oogenesis prior to vitellogenesis show higher resistance to bacterial infection than females that are fully invested in egg production [13]. These observations suggest that investment in egg production could be a direct cost that limits immunity [10].

Natural populations of *Drosophila* are good models to test whether variation in reproductive investment results in difference in immune response, as there is considerable genetic variation for immune performance [14–17], reproductive output [18,19], and potential interaction between the two [7,20]. *D. melanogaster* females in natural populations vary greatly for ovariole number [18,19,21–23] and ovariole number has been reported to be correlated with the number of eggs produced in Drosophila [24,25]. Ovarioles are specialized structures within the *Drosophila* ovary in which oogenesis takes place [26]. The germline stem cells, which are located towards the anterior end of the ovariole, undergo multiple divisions across 14 different stages of development, eventually leading to a mature egg towards the posterior end of the ovariole [26–28]. The number of ovarioles could therefore be a determinant of reproductive investment and could be negatively correlated with immune performance.

To test whether reproductive investment trades off with immune performance in a natural population of *D. melanogaster*, we measured the pairwise phenotypic correlations between ovariole number, egg production, and quality of immune defense across13 isogenic lines from the *Drosophila* Genetic Reference Panel (DGRP) [29], hypothesizing that lines with high ovariole numbers or high egg counts would have lower resistance to infection. However, in our experimental setup, we found no evidence for negative correlation between ovariole number or egg production and immune performance. Furthermore, we found that ovariole number is a genetically robust trait but it was uncorrelated with total egg production and therefore was not a good predictor of reproductive investment.

## Materials and methods

### Fly strains and husbandry

We used 13 different isogenic lines from the *Drosophila* Genetic Reference Panel (DGRP) [29]: RAL-129, RAL-370, RAL-382, RAL-395, RAL-397, RAL-443, RAL-486, RAL-646, RAL-737, RAL-776, RAL-786, RAL-799, and RAL-837 [29]. Based on a prior report [22], lines RAL-382, RAL-395, RAL-397, RAL-646, RAL-776, RAL-786, and RAL-837 were chosen as lines expected to have low ovariole numbers whereas RAL-129, RAL-370, RAL-443, RAL-486, RAL-737, and RAL-799 were chosen as expected to have high ovariole number. The flies were reared at room-temperature (~24°C) with 12:12 h light: dark cycle. Experimental vials were established by placing 10 males and 10 females in a vial containing Cornell cornmeal-sucrose medium (6% cornmeal, 6% yeast, 4% sucrose, 0.7% agar, 0.265% methylparaben, 0.05% phosphoric acid, and 0.5% propionic acid) [30] and allowing them to lay eggs for 2 days. The resulting offspring from each vial were sorted by sex within 1 hour of eclosion, prior to reproductive maturity, and were maintained separately in same-sex pools until they were used for experiments.

### Ovariole count

Ovariole number was determined from 10 females per DGRP line. The females used for ovariole counts were raised in the same experimental conditions as described above. The number of ovarioles in both the ovaries of each female was counted under a light microscope using crystal violet as a staining solution to increase contrast. Mean ovariole number per line was calculated by summing up the ovariole number across both ovaries from each of the 10 flies per line and dividing by 20, which was the total number of ovaries counted for each line (S1 Table in S1 File). Mean ovariole number reported by [22] for each line is also reported in S1 Table in S1 File. We measured the correlation between our current measurement and the measurements reported in [22] using Spearman’s correlation.

### Bacterial infection

Females aged 4–6 days post-eclosion were allowed to mate with males 24 hours prior to infection. The females were then infected with a Gram-negative bacterium, *Providencia rettgeri* [31]. In order to prepare the bacterial suspension, a single colony of *P. rettgeri* was picked from LB agar plate and transferred to a tube containing LB broth. The tube was then incubated overnight with shaking at 37°C. The overnight culture was diluted to A_600_ = 1.0 in sterile phosphate buffered saline (PBS). The flies were infected by pricking the sternopleural region of the thorax with a 0.15 mm diameter metal pin that had been dipped in the bacterial suspension [32]. Control flies were sham-infected by pricking the same location on the body with a sterile pin. The flies were lightly anesthetized with CO_2_ during the infection procedure. In order to make sure the infections were successful and quantify the bacterial inoculum delivered, a subset of flies were homogenized in 1X PBS solution immediately after bacterial infection and were plated on an LB plate and incubated at 37°C overnight. The number of colonies that grew on the plates was used to estimate the infection dose.

### Survival assay

Following infection or sham-infection, females were placed in groups of 10 in fresh vials containing food and were allowed to recover from anesthesia. Flies that failed to recover from anesthesia were discarded from the experiment and survival of the remainder was recorded daily for 5 days. We tested for genetic variability in survivorship using a mixed Cox proportional hazard model with DGRP line as the fixed effect and experimental block as a random effect, implemented in the ‘survival’ package in R [33]. We then compared the survival between DGRP lines using emmeans functions from ‘emmeans’ package (v1.8.3) using the same Cox proportional hazard model. Tukey’s HSD was used for p-value corrections within emmeans.

### Egg and offspring count assay

Five females were kept with eight males in each experimental vial to ensure successful mating. Males were discarded at the time when females were infected. Following infection or sham-infection, females were transferred to new food vials every 24 hours and the eggs laid on those vials were counted every day for 5 days. Once the eggs were counted, the food vials containing eggs were left at room temperature for the flies to develop. Emerged adult flies from each vial were transferred to empty plastic vials 48 hours after the first flies from that vial began to eclose. The adult flies were frozen in the empty vials at −20°C until they were counted.

The study was carried out across three experimental blocks, where each block had three replicate tubes per treatment per DGRP line. For both infected and uninfected treatments, the average number of eggs per female per day was calculated by dividing total number of eggs per tube by total number of females alive within that tube at the beginning of each day. Total egg numbers per female per block were calculated by taking the sum total of eggs across all five days for each DGRP line. We then fitted a linear model with block as a random effect and interaction term between genotype and treatment as fixed effects.


Egg number ~ Genotype x Treatment + Block

We extracted the estimated marginal means adjusted for block from the model using the emmeans() function in R and used those means to carry out correlation studies. Correlation plots and statistics were generated using the ‘ggpubr’ package in R. We further extracted the marginal means of egg production for each treatment condition within each genotype using emmeans(), and then compared the pairwise difference between treatments within each genotype using pairs() function. This function uses Tukey’s adjustment as default. The same analysis procedure was also applied for offspring count.

## Results and discussion

### No correlation between ovariole number and egg output

We first confirmed that the *D. melanogaster* lines we analyzed were variable for ovariole number. The lines ranged from a mean of 12.05 to 27.4 ovarioles per ovary (S1 Table in S1 File). *D. melanogaster* genotype was a highly significant predictor of ovariole number (Levene’s test, *F*_*13, 266*_ = 2.7702, *p* = 0.001). We compared our measurements of mean ovariole number per line to those obtained by [22] for the same lines (S1 Table in S1 File). The measurements were extremely highly correlated (Spearman’s rank correlation test, *ρ* = 0.94, *p* = 4e-07) and the grouping of lines into unambiguous “high” (>20 ovarioles per ovary) and “low” (<15 ovariole per ovary) replicated perfectly across the studies. This indicates that ovariole number is a genetically determined, fixed property of the lines.

**Fig 1:**
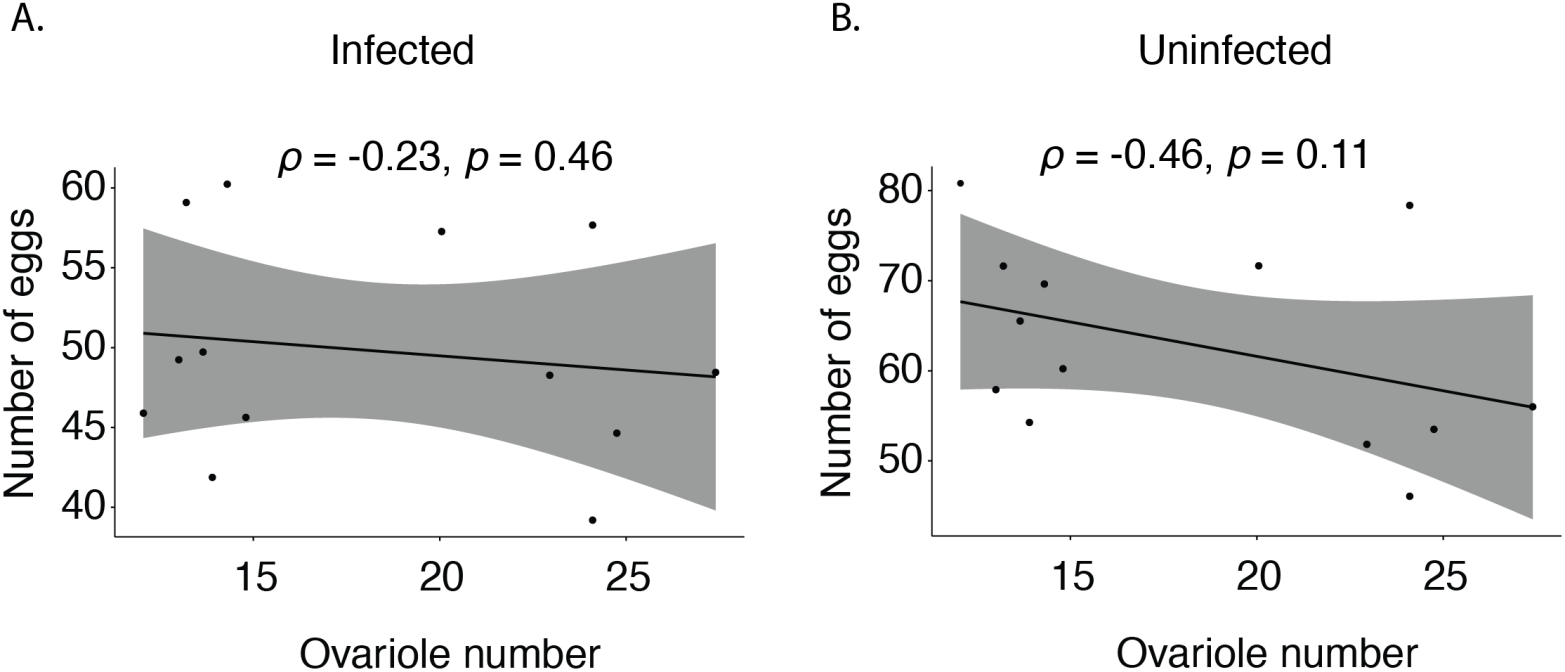
No correlation between ovariole number and egg production across isogenic lines of *Drosophila melanogaster.* Eggs were counted from infected and uninfected females over the course of 5 days (Day 1 to 5 days post-mating). **A)** Spearman correlation between ovariole number and average number of eggs produced by infected females. **B)** Spearman correlation between ovariole number and average number of eggs produced by uninfected females.

We next tested whether ovariole number was predictive of egg production. To our surprise, we found no correlation between ovariole number and egg count either when flies were infected (Spearman’s rank correlation test, *ρ* = −0.23, *p* = 0.46) or when they were sterilely injured (Spearman rank’s correlation test, *ρ* = −0.46, *p* = 0.11) (Fig 1A and 1B). We also tested whether ovariole number is predictive of the number of adult offspring produced, but again found no correlation between ovariole number and offspring per female either when flies were infected (Spearman’s rank correlation test, *ρ* = 0.38, *p* = 0.2) or not (Spearman’s rank correlation test, *ρ* = −0.072, *p* = 0.82) (S1 Fig and S2 Fig in S1 File).

A few principal studies are frequently cited as prior demonstration that ovariole number predicts egg production [18,34–36], although the genetic correlation between the traits is not clear in any of them. David shows a weak positive relationship between ovariole number and fecundity measured in offspring from two contrived crosses [34]. Boulétreau [18] implies a physiological correlation between ovariole number and egg production, showing that both increase proportionally in laboratory-reared females relative to their wild-caught mothers, but it is unclear whether ovariole number and fecundity are genetically correlated in that study. Similarly, Boulétreau et al. [35] indicate that the production rate of eggs from each ovariole is physiologically constant across African and French populations of *D. melanogaster*, and that ovariole number is different between the two populations, but they do not explicitly test for a genetic correlation between fecundity and ovariole number within or across populations. On the other hand, Cohet and David show that ovariole number and egg production are physiologically correlated, but that the rate of eggs produced per ovariole varies with environmental conditions [36]. And Wayne et al. have shown that while ovariole number is a highly genetically variable trait, it does not predict reproductive fitness in a competitive context [23].

Klepsatel et al. have shown that there is a correlation between ovariole number and fecundity in the first few days of adulthood [37], when the highest proportions of eggs are laid [35,38]. In our experimental design, females were aged 4–6 days post-eclosion prior to phenotypic measurement in order to ensure that the fat body was fully mature prior to infection [39], so we cannot rule out the possibility that a correlation between ovariole number and early fecundity could have existed. On balance, it is clear that both egg production and ovariole number are genetically variable within and between populations, and that egg production rate is a physiological function of the number of ovarioles, but realized fecundity is not necessarily genetically correlated with ovariole number.

### No correlation between ovariole number and immune response against *P. rettgeri*

**Fig 2:**
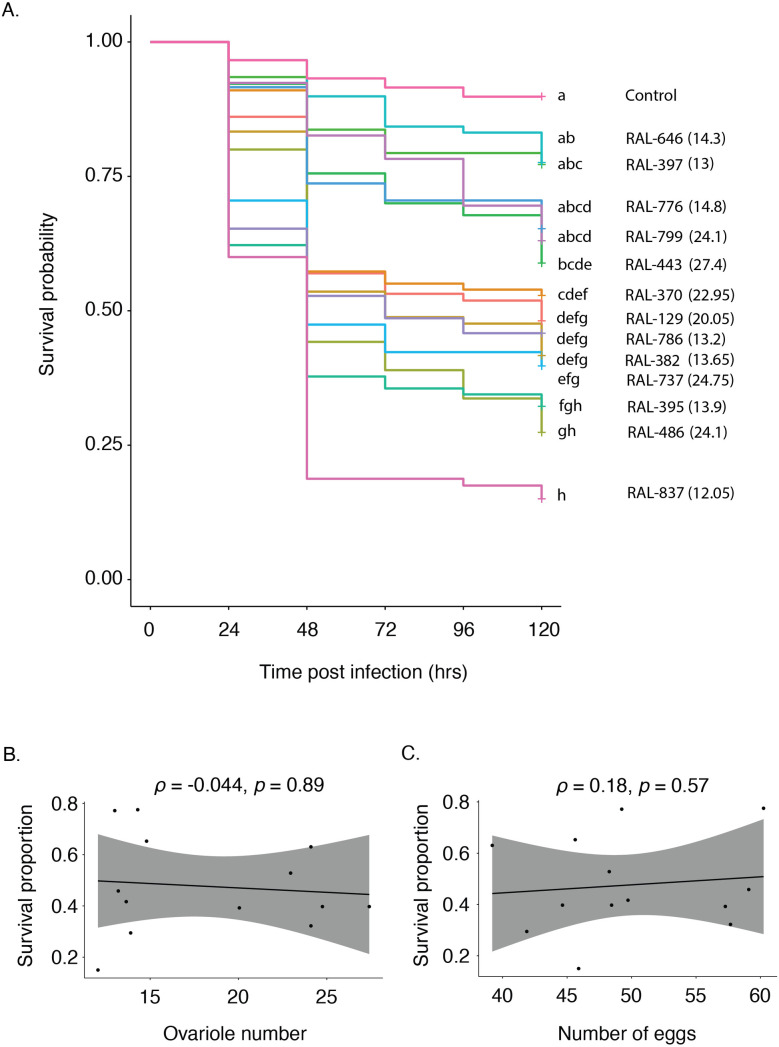
Ovariole number does not predict survival in infected females. **A)** Survival curves for DGRP lines infected with Gram-negative bacteria *Providencia rettgeri*. *D. melanogaster* females were infected with *P. rettgeri* and their survival was tracked for 120 hours post infection. The numbers within the parenthesis indicate the average ovariole numbers for those lines. Survival curves sharing letters are not significantly different from each other. **B)** Spearman correlation between ovariole number and end point survival of infected females. **C)** Spearman correlation between egg production and end point survival in infected females.

Despite the lack of correlation between ovariole number and reproductive output, we tested whether there is a correlation between ovariole number and the survival of females after infection with *P. rettgeri*. The survival proportions for DGRP lines with high and low ovariole numbers were interspersed with each other (Fig 2A), and there was no overall correlation between ovariole number and probability of surviving infection (Spearman’s rank correlation test, *ρ *= −0.044, *p* = 0.89; Fig 2B). The DGRP line with lowest ovariole number (RAL 837) had the highest mortality when infected with *P. rettgeri*. As the genetic correlation in resistance to distinct pathogens can be weak [15], it remains possible that a relationship could exist between ovariole number and resistance to a different pathogen.

### No correlation between egg output and infection survival

**Fig 3:**
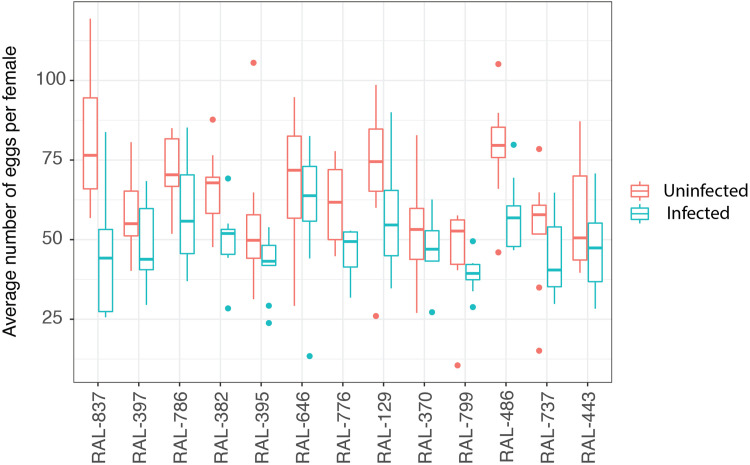
Egg production reduces in infected females. Average number of eggs for each genetic line tested. Eggs were counted from infected and uninfected females over the course of 5 days (Day 1 to 5 days post-mating).

Across genotypes and irrespective of ovariole number, we found a consistent pattern where non-infected flies produced more eggs than infected flies (Fig 3), which could result from energy diversion towards immunity in infected flies [7,10,20]. The difference was significant in lines RAL-837, RAL-382, RAL-776, RAL-129, and RAL-486 (S2 Table in S1 File). Correspondingly, uninfected females produced more adult offspring per female than infected females (S3 Fig in S1 File).

Since egg production was significantly variable among lines even though it was not predicted by ovariole number, we tested whether egg production was negatively correlated with survival of *P. rettgeri* infection, as would be expected if egg production was a direct cost that limited immunity. However, we found no evidence of correlation between the number of eggs produced and survival rate of infected females (Spearman’s rank correlation test, *ρ *= 0.18, *p* = 0.57; Fig 2C). These results are consistent with prior studies showing absence of evolutionary trade-off between reproduction and immunity [40,41]. Such studies may be underrepresented if they are less likely to be carried through to completion by the investigators or published in the primary scientific literature. We note, however, that it remains possible that an evolutionary trade-off could exist between realized infection defense and fecundity within the first few days of adulthood, prior to our study window. If fecundity declines with age [37,35,38], the magnitude of the tradeoff may wane and females may be able to achieve success at both traits.

Interestingly, line RAL-837 exhibited the highest mortality post-infection as well as the largest cumulative reduction in eggs produced after infection. According to life history tradeoff theory, we might expect this line to have the highest survival of infection if active reduction in egg production enabled higher investment in immune response. Contrary to that prediction, though, we see severe post-infection reduction in both survivorship and fecundity in this line. While it could be tempting to conclude that this line is simply “weak” or fragile, it exhibits one of the highest fecundities in the absence of infection and would generally be scored as “healthy” under normal rearing conditions. It is possible that RAL-837 is particularly sensitive to infection and the profound reduction in egg output could be an additional disease symptom. Interestingly, however, the fecundity reduction in this line begins on the second day post-infection, and infected RAL-837 females actually produced slightly more eggs than uninfected females for the first 24 hours post infection (S4 Fig in S1 File). This is distinct from most lines in our study, and is unexpected since a previous study had shown that infection leads to temporary reduction in egg production in the first 72 hours after infection [20]. An alternative possibility is that RAL-837 sustains investment in fecundity despite infection, with a correspondingly impaired immune response during the critical early phase when antimicrobial peptides are being rapidly produced to suppress the infection [42]. This would be consistent with the observed sustained high egg production as well as high mortality within the first 24 hours post infection, and would imply a strong reproduction immunity trade-off with fecundity given priority.

## Concluding comments

Contrary to some published reports but qualitatively consistent with others, our results suggest that ovariole number is not a good predictor of egg number in *Drosophila melanogaster* after infection with *P. rettgeri*, despite the fact that ovariole number is a robustly and repeatably variable genetic trait even when measured in different labs at different times. Furthermore, genotypes with higher ovariole numbers do not necessarily have a reduced defense against bacterial infection, indicating that ovariole number does not trade off with immunity. We also did not find any correlation between the number of eggs produced (realized fecundity) and survival of infection, despite both of these traits being significantly variable across genotypes. Nevertheless, we consistently observed reduction in fecundity after infection and prior studies have documented reductions in immune performance associated with reproductive investment. Consistent with that, we noted one line in our study that may suffer severely compromised immunity correlated with sustained investment in reproduction. Overall, our data suggest that the evolutionary tradeoff between fecundity and survival of infection may be fairly weak – at least under laboratory conditions – even if the physiological tradeoff between them is strong.

## Supporting information

S1 File Supplemental tables and figures. (DOCX)
