# Supplementary material for: Ovariole number does not predict reproductive output or trade-off with immunity in Drosophila melanogaster
Source: PLoS One. 2025 Oct 14;20(10):e0333046. doi: 10.1371/journal.pone.0333046 (PMC12520352; doi:10.1371/journal.pone.0333046)
Supplement: S1 File — (DOCX) [file pone.0333046.s001.docx]

## **Supplemental Tables and Figures**

**Table S1: Average ovariole numbers in DGRP lines used**

| Genotype | Ovariole numbers (our counts) | Ovariole numbers (Lobell et al., 2017) |
| --- | --- | --- |
| RAL 837 | 12.05 | 12.7 |
| RAL 397 | 13.00 | 13.07 |
| RAL 315 | 13.05 | 13.33 |
| RAL 786 | 13.2 | 13.87 |
| RAL 382 | 13.65 | 14 |
| RAL 395 | 13.9 | 13.9 |
| RAL 646 | 14.3 | 14.78 |
| RAL 776 | 14.8 | 15.08 |
| RAL 129 | 20.05 | 28.08 |
| RAL 370 | 22.95 | 24.97 |
| RAL 486 | 24.1 | 25.05 |
| RAL 799 | 24.1 | 24.4 |
| RAL 737 | 24.75 | 25.07 |
| RAL 443 | 27.4 | 29.63 |

**Table S2: Summary statistics for pairwise comparisons between estimated marginal means for treatments within each genotype**

| Genotype | contrast | estimate | SE | df | t.ratio | p.value |
| --- | --- | --- | --- | --- | --- | --- |
| RAL 837 | NI - I | 34.92 | 6.54 | 206 | 5.340 | <0.0001 |
| RAL 397 | NI - I | 8.67 | 6.54 | 206 | 1.325 | 0.1866 |
| RAL 786 | NI - I | 12.54 | 6.54 | 206 | 1.917 | 0.0567 |
| RAL 382 | NI - I | 15.80 | 6.54 | 206 | 2.416 | 0.0165 |
| RAL 395 | NI - I | 12.39 | 6.54 | 206 | 1.895 | 0.0595 |
| RAL 646 | NI - I | 9.40 | 6.54 | 206 | 1.437 | 0.1523 |
| RAL 776 | NI - I | 14.60 | 6.54 | 206 | 2.232 | 0.0267 |
| RAL 129 | NI - I | 14.38 | 6.54 | 206 | 2.199 | 0.0290 |
| RAL 370 | NI - I | 3.57 | 6.54 | 206 | 0.545 | 0.5861 |
| RAL 799 | NI - I | 6.88 | 6.54 | 206 | 1.051 | 0.2943 |
| RAL 486 | NI - I | 20.69 | 6.54 | 206 | 3.163 | 0.0018 |
| RAL 737 | NI - I | 8.86 | 6.54 | 206 | 1.355 | 0.1770 |
| RAL 443 | NI - I | 7.55 | 6.54 | 206 | 1.155 | 0.2492 |

NI - Unifected; I - Infected


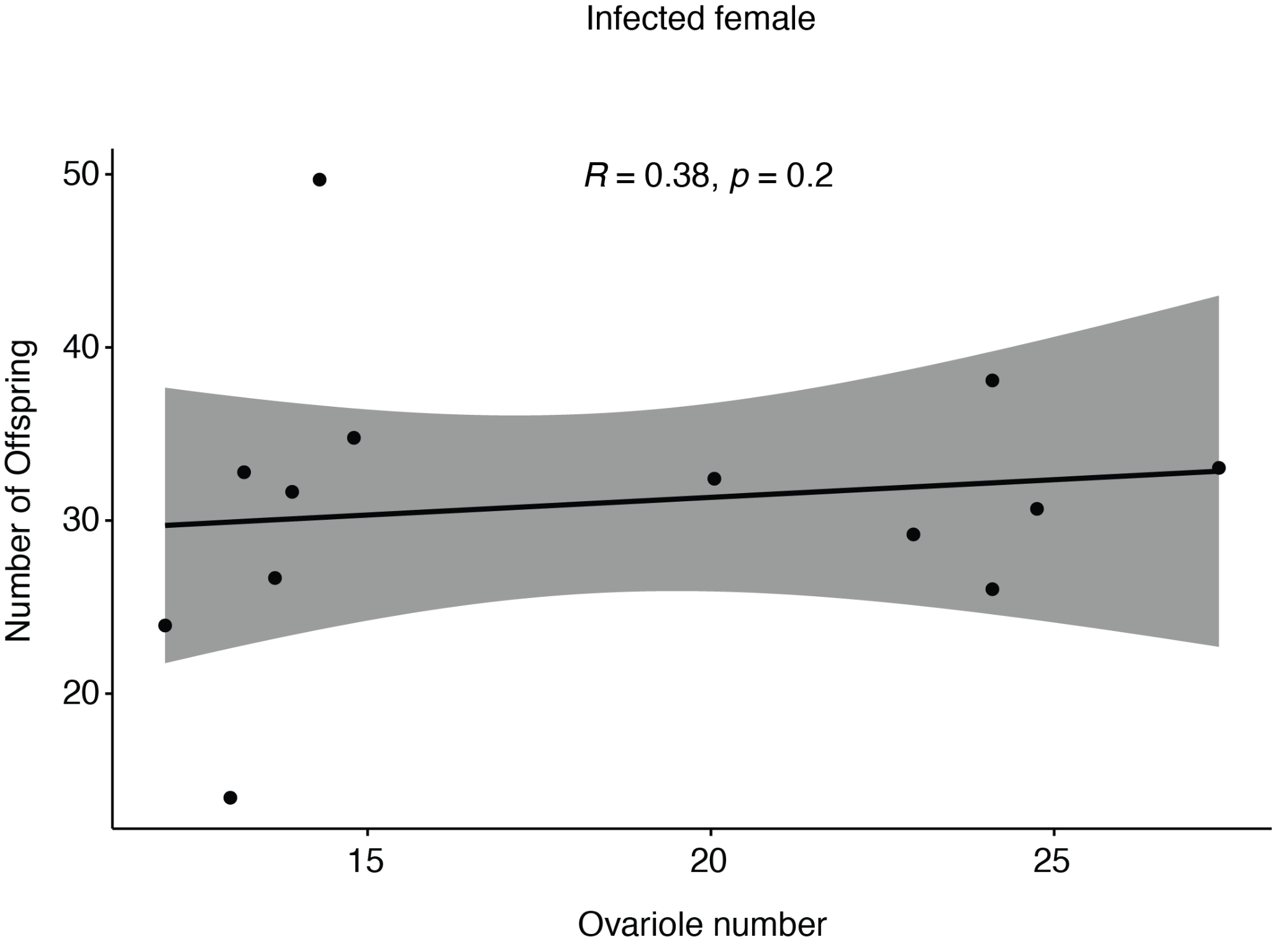


**Fig S1.** Spearman correlation between ovariole numbers and average number of offspring produced by infected females (24 hours to 120 hours post infection). R represents Spearman’s rho.
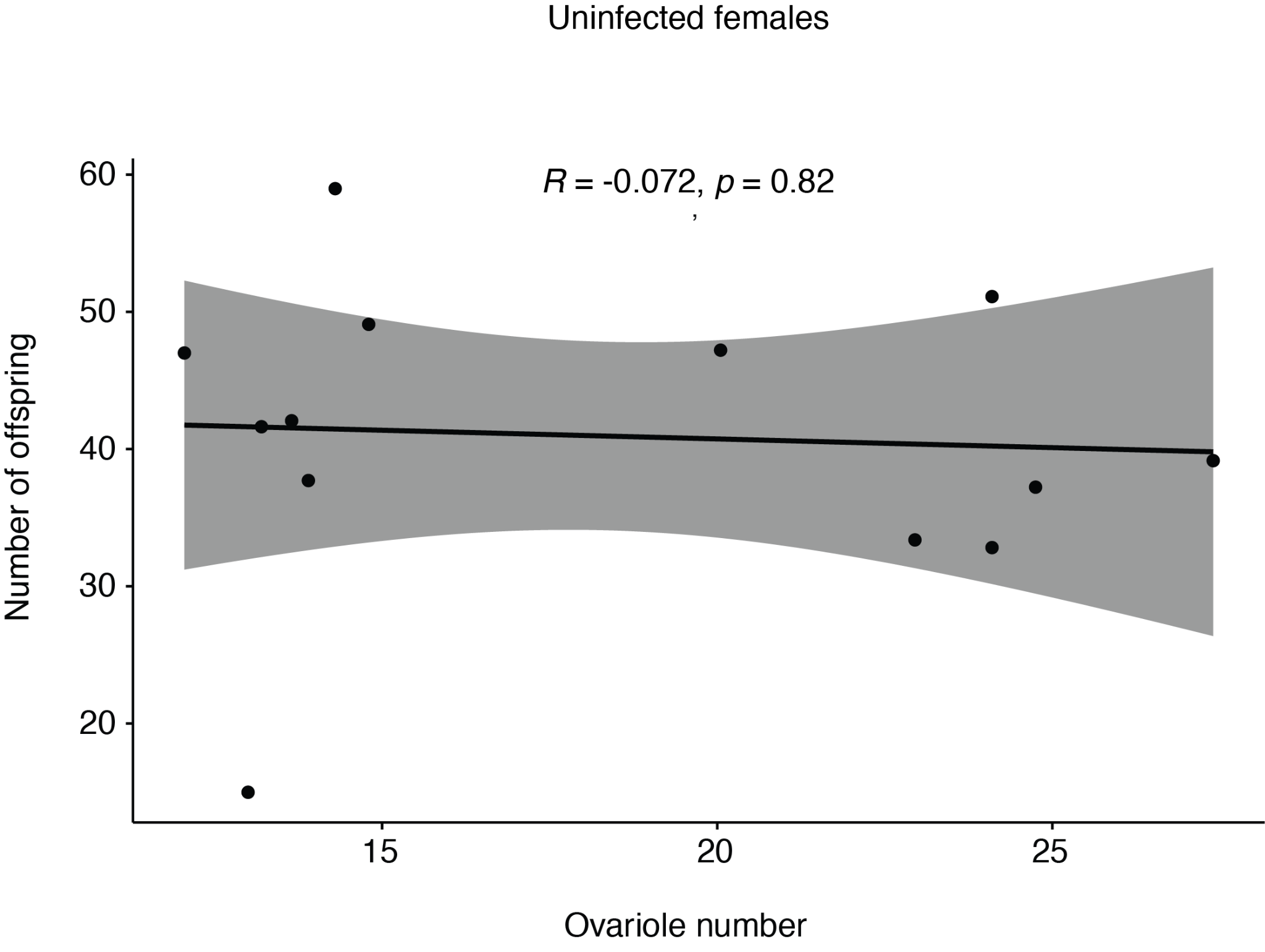


**Fig S2.** Spearman correlation between ovariole numbers and average number of offspring produced by uninfected females (24 hours to 120 hours post infection). R represents Spearman’s rho.


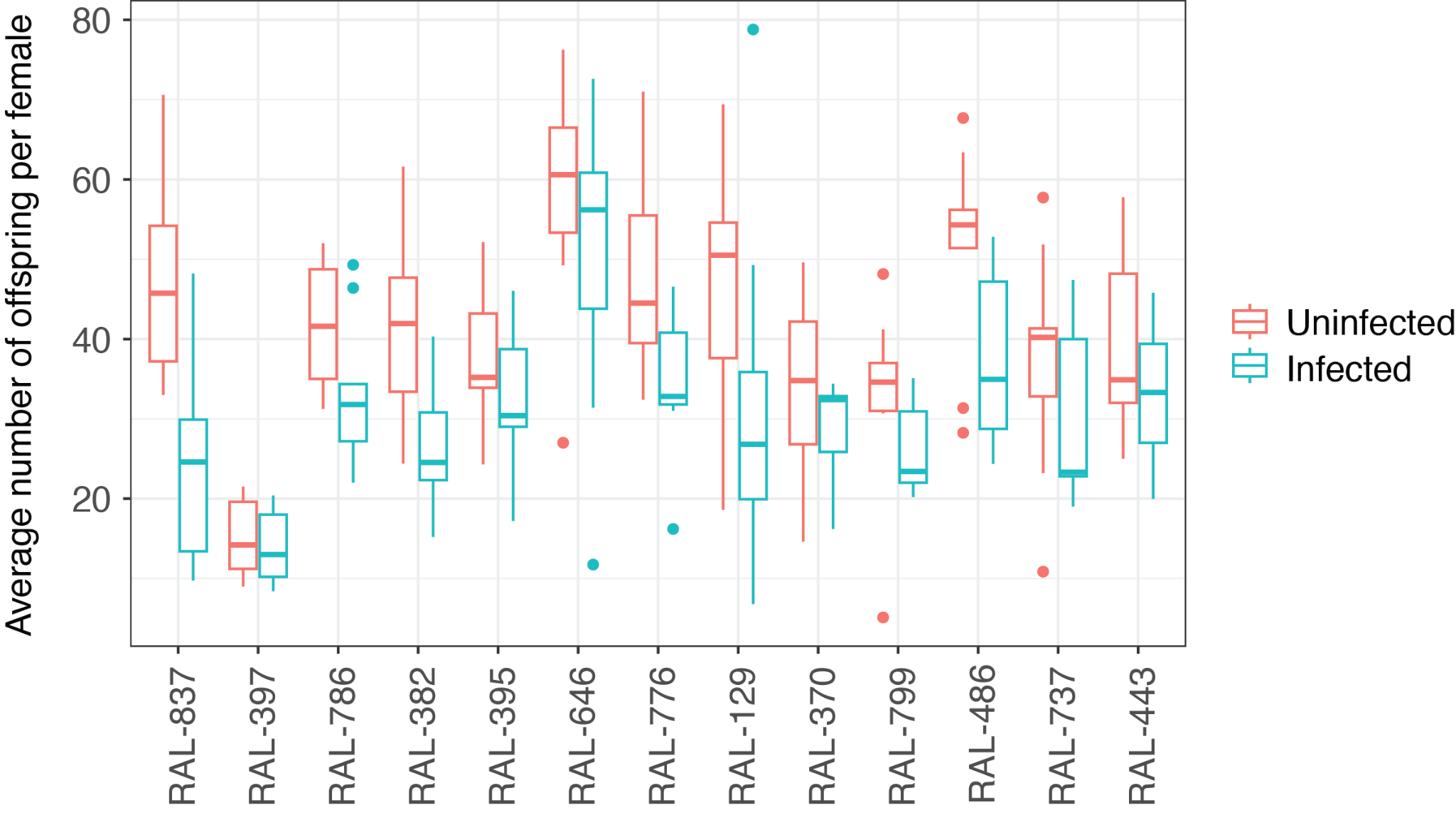


**Fig S3**. Average offspring counts for uninfected and infected DGRP lines from 24 hours to 120 hours post infection.


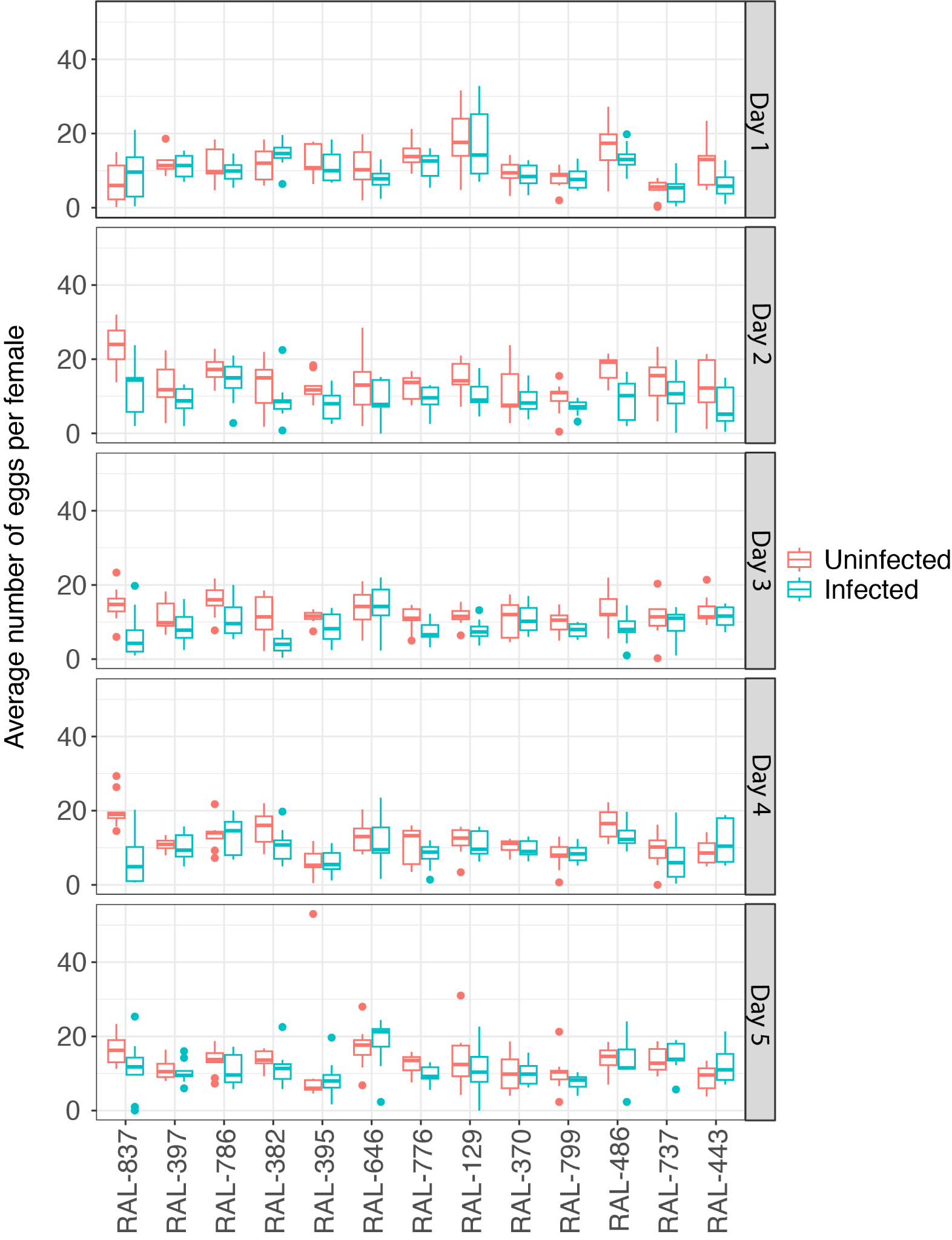


**Fig S4.** Average number of eggs produced by females of each genotype on each day of the study.
